# A Comparative Study of Automated Deep Learning Segmentation Models for Prostate MRI

**DOI:** 10.3390/cancers15051467

**Published:** 2023-02-25

**Authors:** Nuno M. Rodrigues, Sara Silva, Leonardo Vanneschi, Nickolas Papanikolaou

**Affiliations:** 1 LASIGE, Faculty of Sciences, University of Lisbon, 1749-016 Lisbon, Portugal; 2 Champalimaud Foundation, Centre for the Unknown, 1400-038 Lisbon, Portugal; 3 NOVA Information Management School (NOVA IMS), Campus de Campolide, Universidade Nova de Lisboa, 1070-312 Lisboa, Portugal

**Keywords:** prostate cancer, prostate segmentation, prostate detection, deep learning

## Abstract

**Simple Summary:**

Prostate cancer represents a highly prevalent form of cancer worldwide, with timely detection and treatment being crucial for achieving a high survival rate. Manual segmentation, which is the process of manually identifying different anatomical structures or tissues within an image, is the most prevalent detection method. However, it is a time-consuming and subjective task constrained by the radiologists’ expertise, which underpins the demand for automated segmentation methods. In this study, we conduct a comprehensive and rigorous comparison of multiple prevalent deep learning-based automatic segmentation models for the prostate gland and both peripheral and transition zones, using multi-parametric MRI data.

**Abstract:**

Prostate cancer is one of the most common forms of cancer globally, affecting roughly one in every eight men according to the American Cancer Society. Although the survival rate for prostate cancer is significantly high given the very high incidence rate, there is an urgent need to improve and develop new clinical aid systems to help detect and treat prostate cancer in a timely manner. In this retrospective study, our contributions are twofold: First, we perform a comparative unified study of different commonly used segmentation models for prostate gland and zone (peripheral and transition) segmentation. Second, we present and evaluate an additional research question regarding the effectiveness of using an object detector as a pre-processing step to aid in the segmentation process. We perform a thorough evaluation of the deep learning models on two public datasets, where one is used for cross-validation and the other as an external test set. Overall, the results reveal that the choice of model is relatively inconsequential, as the majority produce non-significantly different scores, apart from nnU-Net which consistently outperforms others, and that the models trained on data cropped by the object detector often generalize better, despite performing worse during cross-validation.

## 1. Introduction

Prostate cancer is one of the most common cancer types in the world, affecting roughly one in every eight men according to the American Cancer Society. Despite its high survival rate (5-year relative rates of ≈100% for localized and regional, 30% for distant), it was the third most prominent cancer in 2020 [1]. Therefore, there is an urgent need to develop methods that may aid in early detection and better characterization of disease aggressiveness [2,3]. The latter will make possible the avoidance of over-treatment in patients with non-aggressive disease and the amplification of treatment in patients with aggressive disease. Manual segmentation is still the most common practice, a time-consuming task also limited by the subjectiveness of the radiologists’ expertise, resulting in high interobserver variability.

Most automatic segmentation processes developed for clinical applications are based on convolutional neural networks (CNNs), many being variations of the classic Unet architecture [4]. For prostate segmentation, the proposed models continue to improve as shown by the mean dice score used for the following results. Guo et al. [5] presented a two-step pipeline where a stacked sparse auto-encoder was used to learn deep features, and then a sparse patch matching method used those features to infer prostate likelihood, obtaining a Dice score of 87.8±4.0. Milletari et al. [6] presented Vnet, a volumetric adaptation of unet, achieving a Dice score of 86.9±3.3. Zhu et al. [7] used deep supervised layers on Unet with additional 1 × 1 convolutions, obtaining 88.5 Dice. Dai et al. [8] used the object detection and segmentation model Mask-RCNN, achieving 88±4 and 64±11 Dice in the segmentation of prostatic gland and intraprostatic lesions, respectively. Zavala-Romero et al. [9] presented a multistream 3D model that employed the three standard planes of an MRI volume as inputs and was trained with data from different scanners, achieving Dice scores of 90.5±2.7 for the full gland and 79.9±9.4 for the peripheral zone when using data from one scanner, and 89.2±3.6 and 81.1±7.9 when combining data from both scanners. Aldoj et al. [10] presented a Unet variation with two stacked dense blocks in each level of the downsampling and upsampling paths, obtaining Dice scores of 91.2±0.8, 76.4±2 and 89.2±0.8 for full gland, peripheral and central zones, respectively. Duran et al. [11] presented an attention Unet [12] variation, where an additional decoder was added to perform both gland segmentation as well as multi-class lesion segmentation, achieving a Dice score of 87.5±1.3. Recently, Hung et al. [13] proposed a new take on skip connections, using a transformer to capture the cross-slice information at multiple levels, with the main advantage being that this can be incorporated into most architectures, such as the nnU-Net model.

As of late, more complex and novel architectures and paradigms have been adopted for biomedical imaging segmentation. When dealing with large amounts of data, vision transformer architectures have shown great promise for biomedical segmentation [14,15,16,17,18]. These visions transformer models have the advantage of using self-attention mechanisms, allowing them to learn complex patterns, and to also capture the global spatial dependencies of the images, which is highly advantageous for segmentations. As previously mentioned, since manual segmentation suffers from several limitations, most data end up being unlabeled. Self-Supervised learning strategies have been used to leverage these unlabeled data to improve the performance of segmentation models by first pre-training them on pretext tasks [19,20,21]. Lastly, another paradigm that has been recently used for biomedical image segmentation is knowledge distillation, where models are first trained on a larger task (e.g., multi-centre data ) and are then used to help train other models for more domain-specific tasks [22] (e.g., single-centre data). This technique has already been adopted by some researchers for prostate segmentation [23,24,25] and is shown to improve overall model performance.

One major problem that presents with most previously addressed Unet-based automatic prostate segmentation works are the different evaluation conditions presented, where the same model produces different results depending on the new architecture being proposed and what it needs to outperform the previous models, regardless of the correctness of the experimental settings. What we propose is not a new method but a comparative study of several of the most common, as well as some new some variations, of Unet-based segmentation models for prostate gland and zone segmentation. Additionally, we present and evaluate another research question, regarding the impact of using an object detector model as a pre-processing step in order to crop the MRI volumes around the prostate gland, reducing computational strain and improving segmentation quality by reducing the redundant area of the volumes without simply resizing the data. We examined this question by comparing the results of the different models in both full and cropped T2W MRI volumes, during cross-validation and in an additional external dataset.

## 2. Materials and Methods

### 2.1. Data

We used two publicly available datasets containing retrospective data, one to train both the object detection and the segmentation models (ProstateX), the other to serve as an external test set to assess the quality of the segmentation models (Medical Decathlon prostate dataset). The ProstateX dataset (https://wiki.cancerimagingarchive.net/pages/viewpage.action?pageId=23691656SPIE-AAPM-NCIPROSTATExchallenge, accessed on 2 January 2023) is a collection of prostate MRI volumes that include T2W, DWI and ADC modalities. These volumes were obtained by the Prostate MR Reference Center—Radboud University Medical Centre (Radboudumc) in the Netherlands, using two Siemens 3T MR scanners (MAGNETOM Trio and Skyra). Regarding the acquisition of the images, the following description was provided by the challenge’s organizers: “T2-weighted images were acquired using a turbo spin echo sequence and had a resolution of around 0.5 mm in plane and a slice thickness of 3.6 mm. The DWI series were acquired with a single-shot echo planar imaging sequence with a resolution of 2-mm in-plane and 3.6-mm slice thickness and with diffusion-encoding gradients in three directions. A total of three b-values were acquired (50, 400, and 800), and subsequently, the apparent diffusion coefficient (ADC) map was calculated by the scanner software. All images were acquired without an endorectal coil”.

Regarding the segmentations, the prostate gland segmentations were performed by a senior radiologist from the Champalimaud Foundation, while the transition and peripheral zone segmentations were obtained from the public dataset repository. We applied bias field correction, using N4ITK ([26]), to the T2W volumes, and all the masks were resampled to be on the same space and have the same orientation and spacing as the volumes. A total of 153 volumes were used for the gland segmentation, while for the peripheral and transition zone segmentation, a total of 139 volumes was used.

As an external test dataset, we used the publicly available prostate segmentation dataset from the Medical Segmentation Decathlon ([27]), which was acquired at the Radboud University Medical Centre (Radboudumc) in the Netherlands. This dataset consists of 32 MRI volumes of coregistered T2W and ADC modalities, along with segmentation masks with distinct classes for the transition and peripheral zones. We extracted the T2W volumes and, similarly to the ProstateX, performed bias field correction and resampled the masks, and made new masks by joining both classes to obtain a prostate mask. The model of the scanner used to take these MRIs is not disclosed.

### 2.2. Prostate Detection

**Figure 1:**
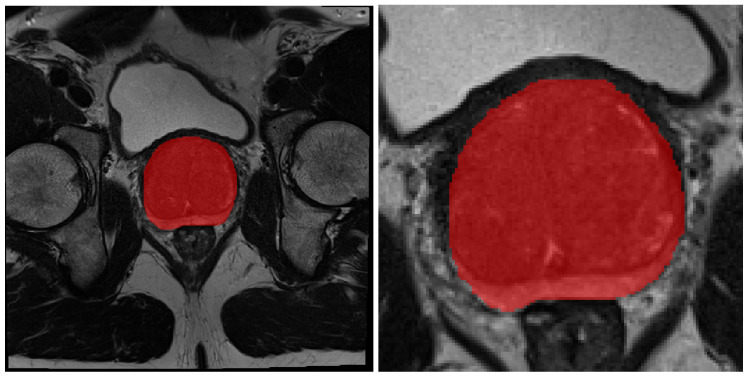
Comparison between original and cropped volumes. Both images correspond to the middle slice of the volumes. The image on the left represents the original full size slice, while the image on the right represents the cropped version.

To prepare the data for object detection, the volumes were converted into 2D 16-bit PNG images (we chose to use 16-bit images so we keep the notion of depth while working in 2D) where the empty slices were discarded. The edge coordinates of the ground truth bounding boxes were obtained from the prostate gland masks. We used these images to train a variation of the YoLo-v4 Open source code available here: https://github.com/ultralytics/yolov5, accessed on 2 January 2023, [28] object detection architecture to locate the prostate gland and accurately draw a bounding box around it (Figure 3). After predicting the bounding boxes, we extracted the coordinates of the edges of the largest bounding box in the volumes, added padding of 40 pixels in each direction, and cropped the entire volume with that box. Using the largest bounding box plus an additional padding, we ensure that all slices contain the entire prostate gland and some additional area. Figure 1 provides a comparison between a standard and cropped slice of a T2W volume.

### 2.3. T2W Pre-Processing and Augmentation

**Figure 2:**
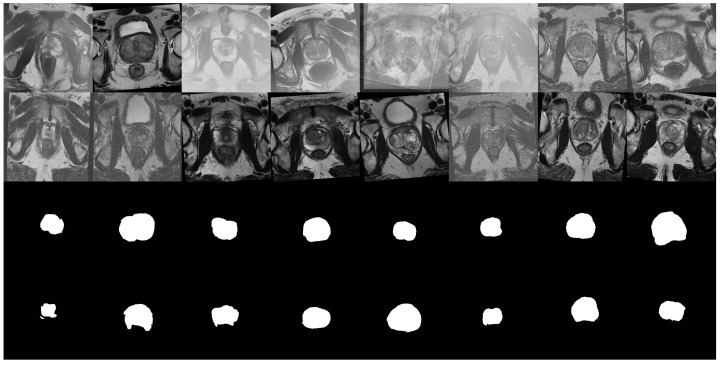
Example of a batch of 16 images, and their respective segmentation masks, from the ProstateX dataset after being pre-processed and augmented. Each image corresponds to the tenth slice of the volume. The images show the effect of the affine transformations as well as the random bias field augmentations.

Before feeding the T2W volumes to the segmentation models, the following pre-processing techniques were applied: Orientation transformation to ensure the models were in RAS+ orientation; Intensity rescaling transformation to ensure the voxel intensity of the volumes was in [−1,1]; *Z* normalization transformation; Cropping the images into a smaller 160 × 160 × 32 size. This last transformation was done to reduce the total unused area, making it less computationally expensive, and to ensure that the volumes had an appropriate number of slices for the segmentation models. It was only applied to the volumes that had not been previously cropped by the object detector. In addition, the following augmentation transformations were applied: Random affine transformations, including rescaling, translation and rotation; Random changes to the contrast of the volumes by raising their values to the power of γ, where γ=0.5; Application of random MRI motion artifacts, and random bias field artifacts. Examples of volumes after pre-processing and augmentation are shown in Figure 2.

### 2.4. Segmentation Models

In this study, we conducted an extensive analysis of various popular Unet-based models from the literature that either had a publicly available implementation or provided enough detail to be reproducible. These models utilized mechanisms falling into three distinct categories: Dense blocks, Recurrent connections, and Attention mechanisms. The inclusion of a diverse range of mechanisms facilitated a thorough investigation of the utility of each mechanism, given that most Unet variations exhibit only minor differences between one another. Additionally, we introduced new networks that build upon the previously published models by incorporating additional combinations of mechanisms. This enabled us to evaluate the performance of these models against established ones, leading to more reliable and informative results.

In total, 13 segmentation models were compared, including: Unet, Unet++ [29], Residual Unet (ResNet), Attention Unet (aunet) [12], Dense Attention Unet (daunet), Dense-2 Unet (d2unet) [10], Dense-2 Attention Unet (d2aunet), Recurrent Residual Unet (r2unet) [30], Recurrent Residual Attention Unet (r2aunet), nnU-Net [31], Vnet [6], highResNet [32], SegResNet [33].

For the Unets, the standard convolution blocks in the downwards path are composed of two convolution operators with a kernel size 3 and stride of 1, followed by a batch normalization, a ReLU activation function and lastly a maxpool operation for dimensionality reduction. The convolution blocks on the upwards path are composed of an upsample operation with scale 2 to double the size of the previous input, and a convolution of kernel size 2 and stride of 1, followed by a batch normalization and ReLU activation.

For the dense unets, the dense blocks are composed of four blocks, with each one having two convolution operations with kernel size 1 and stride of 1, where after each convolution there is a batch normalization and a ReLU activation function. For the transition blocks also used in the dense unets, we perform an upsample operation with scale 2 and a convolution with kernel size 3 and stride of 1, followed by batch normalization and ReLU activation. Regarding the Dense-2 Unet, our implementation differs from the one presented in the original article. As this article does not include enough information to fully replicate the model, we chose the parameters for the dense blocks and convolution blocks to be similar to the ones of the remaining networks.

The recurrent residual blocks are equal to the ones described in Zahangir et al. [30], and are composed of two residual operation and two convolution blocks, each one having one convolution operation with kernel size 3 and stride of 1, followed by a batch normalization and ReLU activation.

The attention mechanisms are equal to those described in Oktay et al. [12], where we calculate $W_{g}$, $W_{x}$ and ψ, each composed of a convolution of kernel size 1 and stride of 1, and then compute $\sigma (\psi \left(ReLU\left(W_{g}+W_{x}\right)\right))$.

At the start of each Unet, a single convolution block, composed of a convolution of kernel size 3 and stride of 1, followed by a batch normalization and ReLU activation, is applied to double the channels from 32 to 64, so we end up with 1024 channels at the bottleneck area of the unets.

Regarding the Vnet, SegResNet and highResNet, we used the models made available by the MONAI package [34]. We used the 3D full resolution version of the nnunet, which is equal to the one described in [31], and publicly available at https://github.com/MIC-DKFZ/nnUNet, accessed on 2 January 2023. All other models were implemented in Pytorch [35].

### 2.5. Training and Evaluation

#### 2.5.1. Detection

The detection model was trained with the ProstateX volumes, which differed in size (320×320, 384×384, 640×640) with a varying number of slices (minimum 19, maximum 27).

All volumes were normalized to zero mean and one unit of standard deviation, resized to 256×256, and separated in 2D slices. The slices with no prostate were removed, ending with a total of 2801 images. The remaining 2801 images were split into 70% training and 30% validation. To avoid data leakage, we ensured all slices belonging to a volume were only present in one of the sets.

To set the initial parameters for the object detector model, we used the genetic algorithm based hyperparameter evolution included in the package, that ran for 100 generations, with 90% mutation and 10% crossover probabilities. The fitness function used to evaluate this evolution is the weighted average between the mean average precision with a threshold of 0.5 (mAP@0.5), which contributed with 10%, and the mean average precision with different thresholds, from 0.5 to 0.95 in steps of 0.05 (mAP@0.5:0.95), which contributed with the remaining 90%. Then, after having set the initial values for the hyperparameters, the model was trained for 350 epochs.

#### 2.5.2. Segmentation

The datasets were split with 5-fold cross-validation. All models except the nnU-Net were trained for a maximum number of 1500 epochs, using early stopping with patience 30. The optimizer was Weighted Adam (AdamW) with a starting learning rate of 1 × 10${}^{-4}$, Cosine Annealing learning rate decay, and weight decay of 4 × 10${}^{-5}$. For nnU-Net, we used the default parameters, training for 1000 epochs with the Ranger optimizer [36]. All models were trained using Pytorch-Lightning.

To assess the quality of the models, we use Mean Dice score (MDS) with 95% confidence interval (CI), Mean Hausdorf distance (MHD) and Mean surface distance (MSD).

The Dice score is a widely used metric for segmentation tasks, and it measures the overlap between the ground truth and predicted masks. The higher the score, the better the segmentation.

$$DS\left(X,Y\right)=\frac{2|X\cap Y|}{\left|X|+|Y\right|}$$

The Hausdorff distance (HD) measures how far two points are in two images. In this case, how far a point from the predicted mask if from its nearest point in the ground truth mask, essentially indicating the largest segmentation error present in the predicted mask. Low values mean small errors.

$$HD\left(X,Y\right)=\max_{x\in X}\min_{y\in Y}||x-y||$$

The Surface distance (SD)measures the difference between the surface of the predicted mask and the ground truth mask. Low values mean small differences.

$$SD\left(X,Y\right)=\min_{x\in X,y\in Y}||y-x||$$

### 2.6. Loss Function

Initially, our loss function was the standard averaged sum of Dice (Equation (2)) and Crossentropy (Equation (1)), called Dice CE loss (Equation (3)). It produced good results for the mid regions of the prostate, but struggled with the small and irregular shapes present in both apex and base. Therefore, we included both Focal (Equation (4)) ([37]) and Tversky (Equation (5)) losses, as they mitigate this issue by focusing on the hard negative examples, reducing both false negatives and false positives. The final loss function was named Focal Tversky Dice Crossentropy loss (FTDCEL) (Equation (6)), an averaged sum of all previously mentioned loss functions, all having the same weight (FTDCEL) (Equation (6)). All of these loss functions were implemented using the MONAI package [34].

(1) $$CE=\left\{\begin{array}{cc} -log(p) & \;\mathrm{if}\;y=1\\ -log(1-p) & \;\mathrm{otherwise} \end{array}\right.$$

(2) $$DL\left(p,\hat{p}\right)=\frac{2p\hat{p}+1}{p+\hat{p}+1}$$

(3) $$DCE\left(p,\hat{p}\right)=\frac{DL\left(p,\hat{p}\right)+CE\left(p,\hat{p}\right)}{2}$$

(4) $$\begin{array}{c} \mathrm{rewriting}\;\mathrm{CE}\;\mathrm{as}\;p_{t}=\left\{\begin{array}{cc} p & \;\mathrm{if}\;y=1\\ 1-p & \;\mathrm{otherwise} \end{array}\right.\\ FL\left(p_{t}\right)=-\alpha_{t}{\left(1-p_{t}\right)}^{\gamma }log\left(p_{t}\right) \end{array}$$

(5) $$TL\left(p,\hat{p}\right)=1-\frac{1+p\hat{p}}{1+p\hat{p}+\beta \left(1-p\right)\hat{p}+\left(1-\beta \right)p\left(1-\hat{p}\right)}$$

(6) $$FTDCEL\left(p,\hat{p}\right)=\frac{DCE\left(p,\hat{p}\right)+FL\left(p,\hat{p}\right)+TL\left(p,\hat{p}\right)}{3}$$

## 3. Results

### 3.1. Object Detection

**Figure A1:**
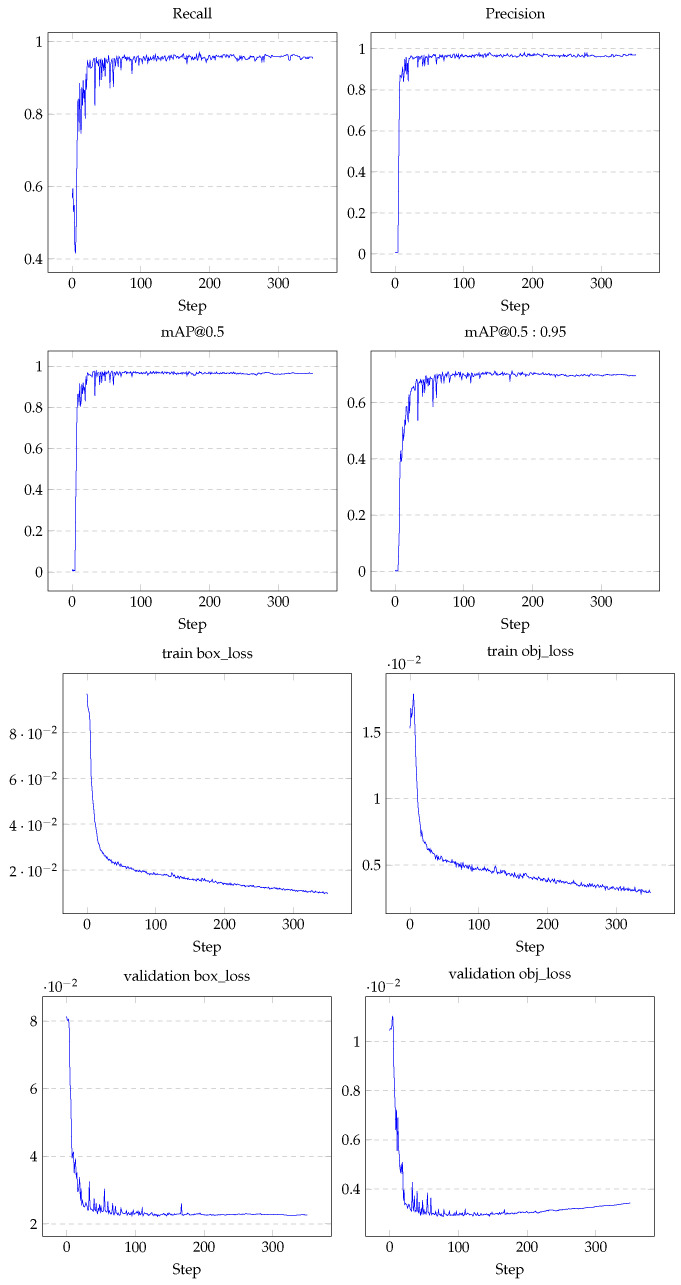
Evolution of the different metrics during the training of the prostate detection model. The figure contains the Recall, Precision, mAP@0.5, mAP@0.5:0.95, and both the object and box loss for the training and validation sets.

To evaluate the object detection model we followed the procedure described in Section 2.5.1 for four different variations of the yolo model, a small, medium, large and extra-large. Figure A1 shows the results obtained on the medium model which is the one that produced the best results.

**Figure 3:**
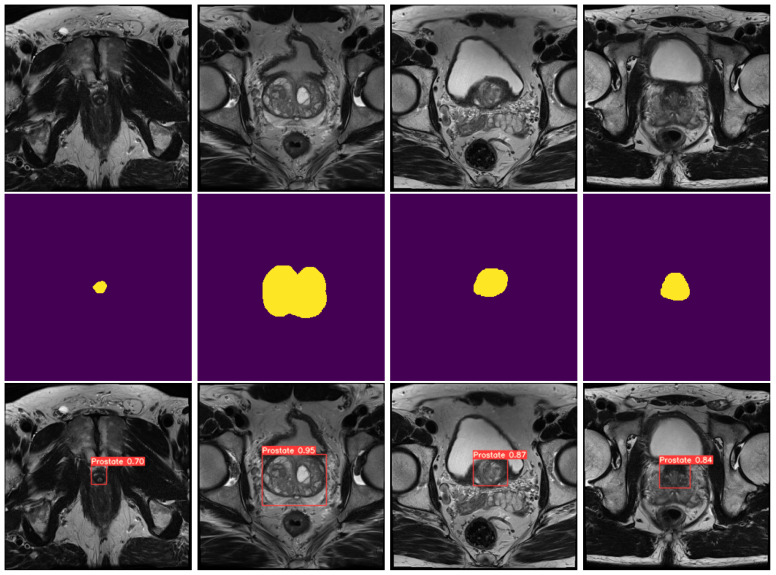
Comparison between original prostate images, masks and predicted volumes. The top row consists of random slices from different volumes, the second row consists of the respective prostate masks, and the third row consists of the respective predicted bounding boxes, with confidence value of the prostate on each of the images.

As shown in Figure A1, the results obtained were very accurate, with high confidence values on all prostate sections, including the apex, achieving a Precision, Recall, mAP@0.5 and mAP@0.5:0.95 of 0.9709, 0.9534, 0.9653 and 0.6965, respectively. As for the loss values, which represent the error meaning lower values are better, this model obtained a bounding box loss (Box loss), which is the measure of how well the predicted bounding box covers the target, of 0.0227 and an object confidence loss (Obj loss), which measures the probability that the target exists inside the predicted bounding box, of 0.0034, on the validation data. Figure 3 (bottom row) illustrates a batch of the obtained results. As shown, both the apex, base and middle area of the prostate is properly detected with a high degree of confidence.

### 3.2. Gland Segmentation

**Figure 4:**
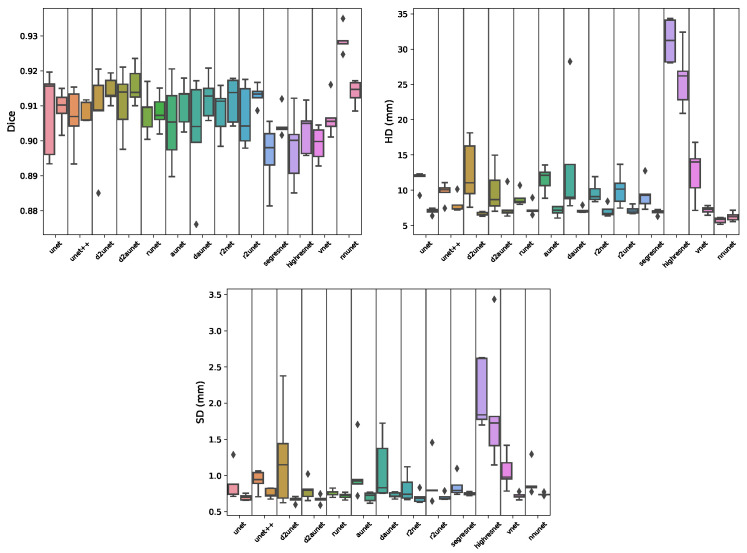
Boxplots showing the distribution of Dice, Hausdorff distance (HD) and Surface distance scores per model during cross-validation for the gland segmentation task. Each segment contains a pair of boxplots, where the left one corresponds to the results of the model on the full data, and the right one on the cropped data.

Regarding the segmentation of the prostate gland (Table 1A and Figure 4), when working with the full volumes we can see that the nnunet is significantly better than all other models, by ≈2%, achieving a mean Dice score (MDS) of 0.9289±0.0046. This is further corroborated when looking at the boxplots as we can see that most models have a high degree of dispersion, as opposed to the nnunet. A mean Hausdorff distance (MHD) of 5.7155 is considerably better than the ones obtained by the remaining models. It can also be observed that despite small differences, there is no statistical difference between the Dice score results obtained by the other models, although being discernible that some, such as daunet, d2uNet, Vnet and especially the highresnet, have substantially greater MHD values. From Figure 1 right we can see that nnunet is the model that better generalizes, achieving a MDS of 0.8678 on the external test set, a result far greater than most other models, along with a MHD of 10.0231, which is the smallest by far between all models.

When segmenting the prostate gland using the volumes cropped by the object detection model (Figure 1 left and Figure 4), the results are quite different. There is no single significantly best model, there are however four models that under-perform, and nine which have similar scores. The best, although not significantly better, Dice score is obtained by the d2auNet, with a value of 0.9158±0.0068. Looking at the boxplots we can see that the majority of the models, apart from the r2Net and highResNet, present a low degree of dispersion. Looking at the MHD and average surface distance (ASD), we can further see that the choice of the model is mostly inconsequential, with the exception of highresnet. These results still hold for the external test set (Figure 1 right), where we can see that all models, again with the exception of highresnet, have a very similar generalization capability with a MDS of ±0.85. Comparing the performance of full and cropped volume models, looking at Table A1 it is possible see that for 11 of the 13 models there is no significative difference, with the only two exceptions being the Vnet, where it does in fact improve the Dice scores, and the nnU-Net which has the opposite effect, worsening model performance. Looking at the boxplots in Figure 4, we can see that on all metrics the results show less dispersion, which is a good indication. However, when observing the difference between the results on the external test set, we can see that the vast majority of models do improve their generalization capability using cropped data, achieving higher MDS scores and lower MHD scores.

**Figure 5:**
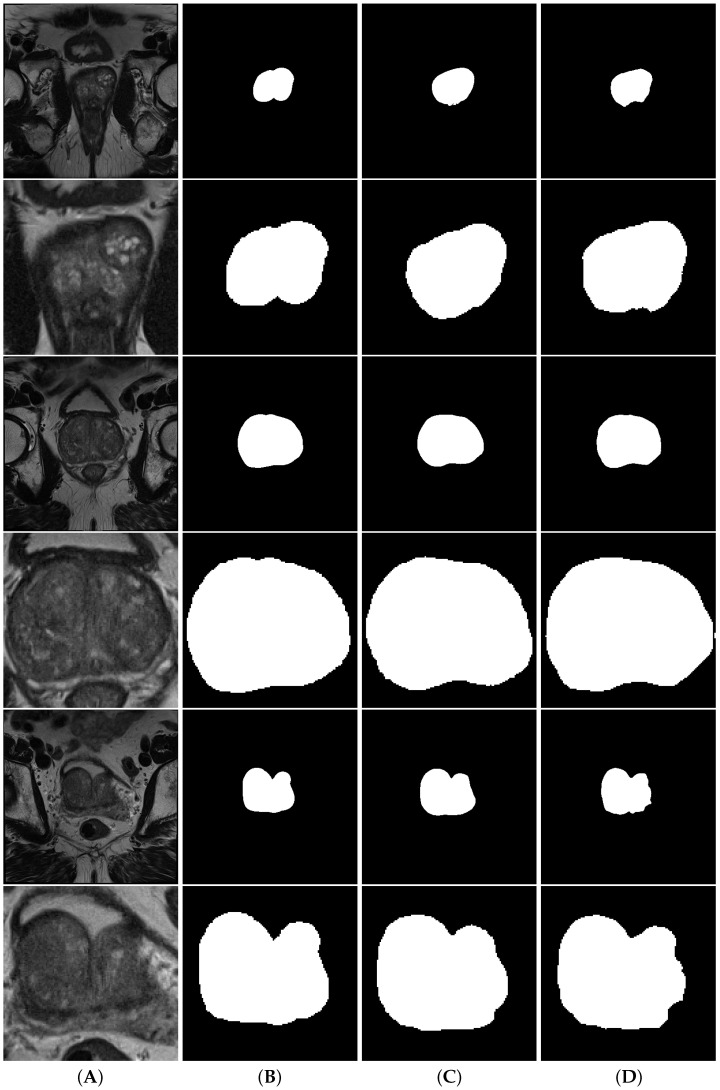
Segmentations of the prostate gland. Column (**A**) contains the volumes, column (**B**) contains the ground truth, column (**C**) contains the nnU-Net segmentations and column (**D**) contains the d2aunet segmentations. Rows are interleaved, showing a full volume and a model cropped by the object detection model, respectively.

Figure 5 shows a comparison between full volume and cropped volume segmentations of both nnU-Net and d2aunet, as they were the models with the highest Dice score in each task. In this case, despite having a lower MDS, the segmentations provided by the cropped models are arguably better than those provided by the full volumes, with the difference being explained by the difference in size when calculating this metric. In smaller volumes, a wrongly calculated pixel will have more impact in the calculation of the MDS than on larger volumes, which may explain why a model with a slightly lower MDS (0.014 difference) can provide more accurate segmentations.

### 3.3. Zone Segmentation

**Figure 6:**
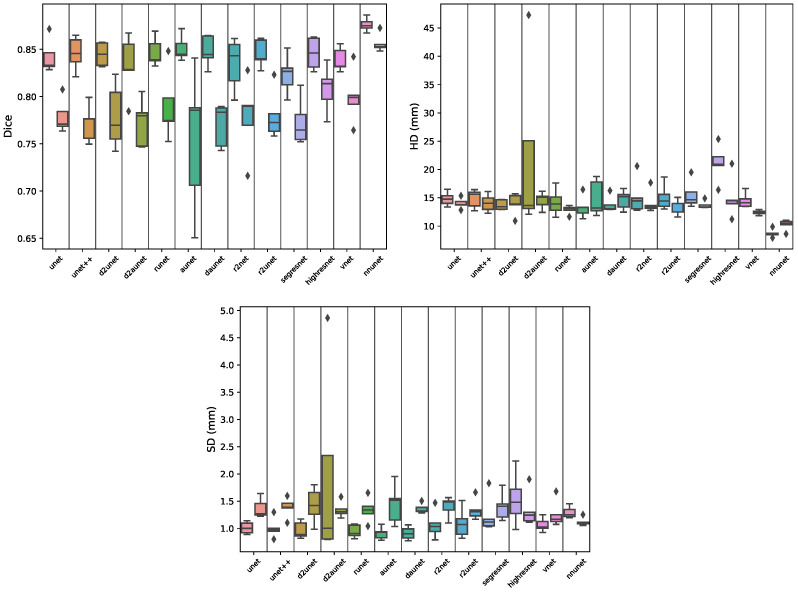
Boxplots showing the distribution of Dice, Hausdorff distance (HD) and Surface distance scores per model during cross-validation for the transition segmentation task. Each segment contains a pair of boxplots, where the left one corresponds to the results of the model on the full data, and the right one on the cropped data.

Starting with the transition zone (TZ) segmentation (Table 2A and Figure 6), we can see that the nnU-Net outperformed all the other models, both on the full volume and cropped volume modalities. On the full volume data, it achieved an MDS of 0.8760±0.0099, which is ≈3% better than all other models, while on the cropped data it further increased the distance to the remaining models, by achieving a mean Dice score of 0.8561±0.0133, ≈7% better than the others. Regarding the maximum error, for full and cropped we obtained an MHD distance of 8.7049 and 10.2390, respectively, which are notably smaller than the errors obtained by the remaining models, ±6 and ±4 mm smaller for the full and cropped volumes, respectively. However, when the models are evaluated on an external test set (Figure 2), we can see that these results do not hold. For the full volume data the nnU-Net falls short, dropping ≈15%, while most other variations of the Unet dropped far less, with the regular Unet being the model with the best MDS of 0.77, despite having a larger maximum error. On the cropped volumes, the nnU-Net remained the best performing model, with an MDS of 0.7540.

**Figure 7:**
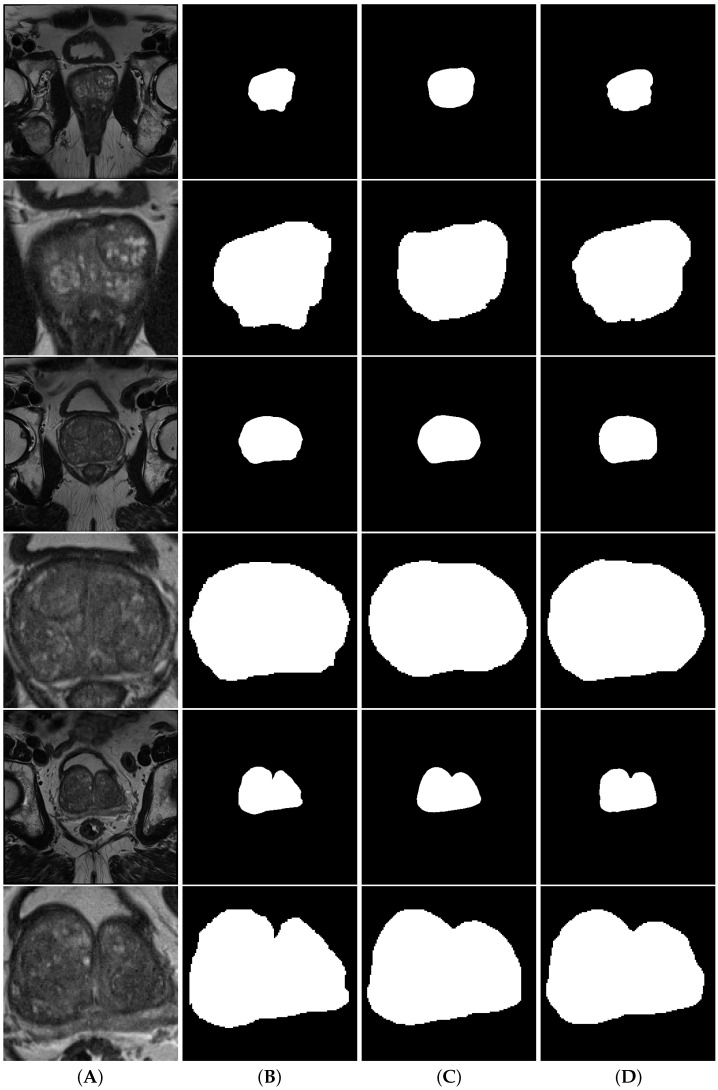
Segmentations of the transition zone. Column (**A**) contains the volumes, column (**B**) contains the ground truth, column (**C**) contains the nnunet segmentations and column (**D**) contains the aunet segmentations. Rows are interleaved, showing a full volume and a model cropped by the object detection model, respectively.

When comparing the performance of full and cropped volume models, looking at Table A2 and Table 2, it is clear that the performance of all models drops significantly when using the cropped data. One interesting particularity to note is that while in the majority of the models, the dispersion of the results on the cropped data is similar to the dispersion on the full data, when observing d2aunet we can see that dispersion of values for both the Hausdorff and surface distances is greatly reduced (Figure 6). Figure 7 shows a comparison between full volume and cropped volume segmentations of both nnU-Net and aunet, as they were the models with the highest Dice scores during cross-validation.

**Figure 8:**
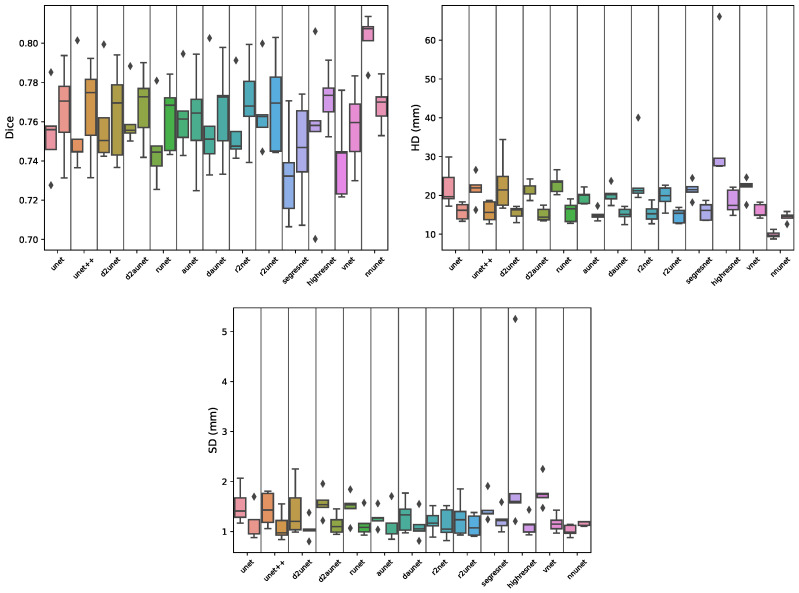
Boxplots showing the distribution of Dice, Hausdorff distance (HD) and Surface distance scores per model during cross-validation for the peripheral segmentation task. Each segment contains a pair of boxplots, where the left one corresponds to the results of the model on the full data, and the right one on the cropped data.

Regarding the peripheral zone (PZ) segmentation (Figure 3 and Figure 8), when working with the full volumes we can see that the nnU-Net outperformed all other models, achieving an MDS of 0.8029±0.0063, ≈5% better than the remaining models, but also the smallest error, with an MHD of 9.8693, less than half of the average of most other models. When working with the cropped volumes, we can see that there is no statistically significant difference between any of the models. They all produce very similar values for all evaluated metrics (MDS, MHD, ASD). Looking at Figure 8, we can also see that the distribution and dispersion are very similar, with only a few outliers such as the aunet when measuring the HD and the d2unet when measuring the SD. When testing the models on the external test set (Figure 3), we can see that while using the full volumes the results hold, with nnU-Net still being the top performing model with an MDS of 0.6835 and having the lowest MHD of only 13.4527. For the cropped volumes, the d2unet was the best-performing model, with an MDS of 0.6387, and the lowest MHD of 16.3358.

**Figure 9:**
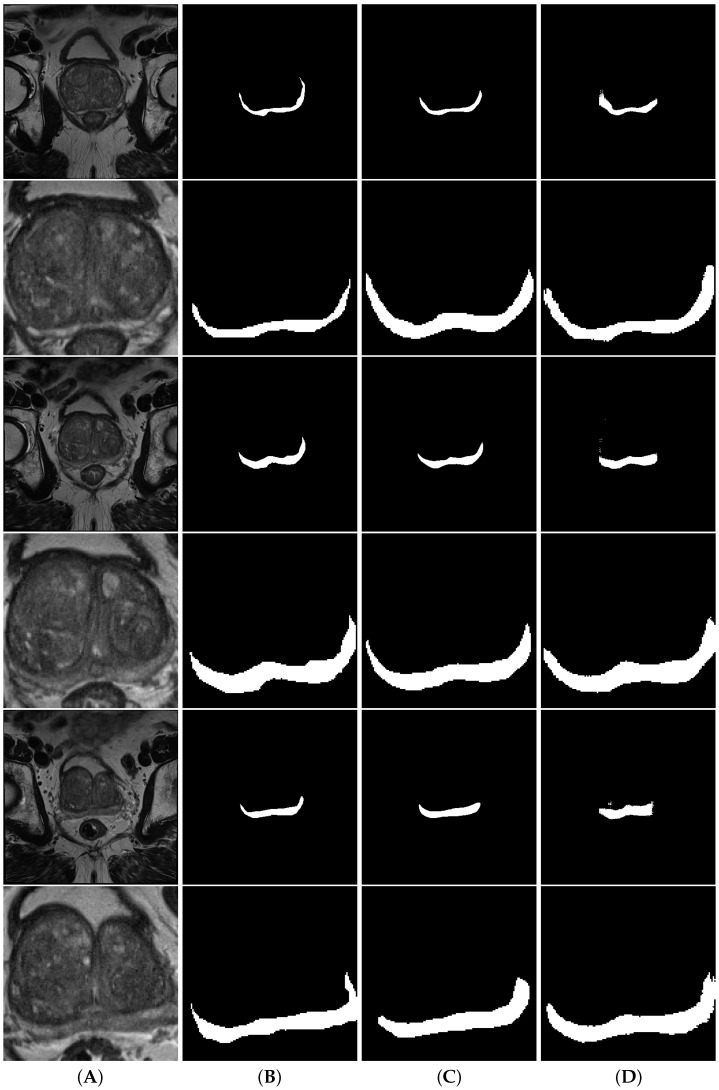
Segmentations of the peripheral zone. Column (**A**) contains the volumes, column (**B**) contains the ground truth, column (**C**) contains the nnU-Net segmentations and column (**D**) contains the highResNet segmentations. Rows are interleaved, showing a full volume and a model cropped by the object detection model, respectively.

When comparing the performance of full and cropped volume models, looking at Table A3 and Table 3, we can see that there is no statistical difference between using full or cropped data when segmenting this zone, apart from the nnU-Net that similarly to what was shown in the two previous zones performs significantly worse. However, similarly to what was observable on the gland analysis, we can see that many of the models show small improvements on the external test set, most noticeably when examining the MHD values, showing again that cropping may improve the generalization capabilities. Figure 9 shows a comparison between full volume and cropped volume segmentations of both nnU-Net and highResNet, as they were the models with the highest Dice score during cross-validation. When looking at the full volume segmentations, we can observe a large difference between the nnU-Net and the highResNet. While the first produces smooth and well-defined segmentations, the latter shows clear signs of poorly defined edges and obvious noise in some cases.

## 4. Discussion

We conducted an extensive analysis of several commonly used segmentation models for prostate gland and zone segmentation on a unified pipeline with the same settings for all. In addition, we answered the research question regarding the effectiveness of using an object detector to crop the MRI volumes around the prostate gland to aid during the segmentation process.

First, we trained an object detector model to perform bounding box detection on the prostate gland, in order to later crop the images. We employed a variation of the Yolo-v4 model to detect the prostate on 2D slices, which provided very good results, with box and confidence validation losses of 0.0227 and 0.0034, respectively. These results show that it is fairly easy to train a robust prostate detection model, raising the hypothesis for a feature work of leveraging the learned local representations of such a model and using them to aid in a segmentation task.

Using the results obtained from the object detection, we produced a new version of the ProstateX dataset where the volumes were cropped around the gland, reducing the overall size of the image and computational power required. We then trained 13 different commonly used segmentation models, with some additional new variations, on both the full and cropped volumes. The models were subsequently validated on the prostate dataset from the medical imaging decathlon, both on the original and on a cropped version.

When comparing the performance of the models on the full volume segmentation tasks, two main conclusions can be drawn. The first being that the nnU-Net is the overall best model, outperforming all other during cross-validation on the three different segmentation tasks, while still being the model that better generalized on the external test set, on two out of three tasks. Interestingly, despite not being the worst performing model on the external transitional data, it was the one with the highest ASD value, meaning that while it did not make the largest mistakes, it did make the most mistakes out of all models. The second conclusion is that, excluding the nnU-Net, when choosing between the other models, the decision is almost inconsequential. While SegResNet and Vnet produced results significantly worse than some other models, the remaining show no significant differences between each other for the three segmentation tasks.

When comparing the performance of the models on the cropped volume segmentation tasks, the results are not as clear as when using the full volumes. One common aspect among all three segmentation tasks is that the nnU-Net is either the best-performing model, or is at least one of the best. While for the transitional segmentation task, the nnU-Net is the clear winner, for both the gland and peripheral segmentation tasks, the choice of the model is almost inconsequential, with the exception of only four models on the gland task, which perform significantly worse. Regarding the performance on the external test set, there is no clear indication of an overall better model. While for the transition segmentation task, the nnU-Net is clearly better than the other models, and for the remaining two tasks, the results are very similar.

Lastly, comparing the performance of the models when using the two types of data, it is observable that overall the model performance during cross-validation is either the same, or statistically significantly worse. The clearest case being the nnU-Net, where it consistently hinders performance. This is most likely due to the way nnU-Net processes the data, since it is based on a set of heuristics that are applied based on the characteristics of the data, characteristics that are changed when the volumes are cropped. However, in both the gland and peripheral zone segmentation task, it is also observable that the cropped models generalize better, despite their poorer performance during cross-validation, hinting that it is worthwhile to further explore this approach, ideally on a larger dataset and with data to rule out any hypothesis regarding data quantity.

## 5. Conclusions

In this paper, we perform a comparative study using several of the latest, commonest, and certain variations of Unet-based segmentation models for prostate gland and zone segmentation. We answer the research question regarding the impact of using an object detector model as a pre-processing step in order to crop the MRI volumes around the prostate gland. Regarding the comparison of the different architectures, it is clear that overall there are statistically significant differences between the vast majority of models, regardless of the mechanisms they employ, apart from the nnU-Net model [31], which consistently outperforms all other models. Concerning the object detector, it is shown that despite being straightforward to train and obtain good results for prostate detection, overall the segmentation results are the same or statistically significantly worse.

## Figures and Tables

**Table 1:** (**A**) Results obtained by the segmentation models on prostate gland segmentation task. The results presented are the mean scores from the validation data over the five-fold models. The best results (based on Table A1) for both the full and cropped volumes are presented in green. (**B**) Results obtained on the external test set by the segmentation models trained on the prostate gland segmentation task. The results presented are the mean scores of the 5-fold models over all the samples on the external test set. The best results for both the full and cropped volumes are presented in red.

(**A**)								
	Gland				Cropped Gland			
	MDS	CI	MHD (mm)	ASD (mm)	MDS	CI	MHD (mm)	ASD (mm)
unet	0.9082	0.0154	11.5690	0.8732	**0.9094**	0.0064	7.0381	0.6990
unet++	0.9066	0.0108	9.7262	0.9289	0.9081	0.0037	7.9955	0.7533
runet	0.9081	0.0078	8.8370	0.7610	**0.9083**	0.0062	7.3480	0.7186
aunet	0.9052	0.0151	11.5549	1.0364	**0.9105**	0.0079	7.0760	0.7047
daunet	0.9023	0.0203	13.5009	1.0892	**0.9123**	0.0076	7.1538	0.7265
d2unet	0.9078	0.0170	12.5156	1.2563	**0.9145**	0.0047	6.6846	0.6664
d2aunet	0.9110	0.0115	9.9763	0.7997	**0.9158**	0.0068	7.6787	0.6715
r2unet	0.9084	0.0087	9.6704	0.8259	**0.9117**	0.0081	7.0649	0.6994
r2aunet	0.9069	0.0110	10.1465	0.8990	**0.9130**	0.0036	7.1827	0.7096
vnet	0.8991	0.0062	12.5448	1.0621	0.9067	0.0070	7.2024	0.7220
segresnet	0.8960	0.0117	9.3760	0.8540	0.9048	0.0051	6.9018	0.7490
highresnet	0.8980	0.0130	31.1897	2.1118	0.9029	0.0084	25.8385	0.7533
nnunet	**0.9289**	0.0046	5.7155	0.9218	**0.9139**	0.0044	6.2565	0.7406
(**B**)								
	Gland				Cropped Gland			
	MDS	MHD (mm)	ASD (mm)		MDS	MHD (mm)	ASD (mm)	
unet	0.8474	16.9045	2.3177		**0.8595**	9.9819	1.2044	
unet++	0.8544	25.7024	2.0092		0.8472	9.0465	1.3394	
runet	0.8501	19.8740	1.9686		0.8547	9.9693	1.2591	
aunet	0.8291	25.3664	2.5814		0.8521	9.9022	1.3618	
daunet	0.8277	21.4430	2.1456		0.8579	10.6797	1.2818	
d2unet	0.8528	15.2098	1.7646		0.8567	9.1642	1.3421	
d2aunet	0.8464	13.6260	1.9367		0.8562	8.2389	1.3334	
r2unet	0.8517	14.9619	1.7727		0.8546	8.7872	1.3819	
r2aunet	0.8514	16.2630	1.9737		0.8573	10.3648	1.7792	
vnet	0.8368	21.3305	2.7043		0.8548	9.6195	1.3882	
segresnet	0.8479	17.4913	1.7528		0.8468	9.7496	1.3192	
highresnet	0.7448	69.6783	11.5506		0.8092	35.0037	3.6740	
nnunet	**0.8678**	10.0231	3.3704		0.8558	9.2565	1.6221	

**Table 2:** (**A**) Results obtained on the external test set by the segmentation models trained on the transitional zone segmentation task. The results presented are the mean scores of the 5-fold models over all the samples on the external test set. The best results (based on Table A2) for both the full and cropped volumes are presented in green. (**B**) Results obtained by the segmentation models on transitional zone segmentation task. The results presented are the mean scores from the validation data over the five folds. The best results for both the full and cropped volumes are presented in red.

(**A**)								
	Tz				Cropped Tz			
	MDS	CI	MHD (mm)	ASD (mm)	MDS	CI	MHD (mm)	ASD (mm)
unet	0.8423	0.0243	14.0153	1.0092	0.7790	0.0250	14.0153	1.3668
unet++	0.8457	0.0254	14.0649	1.0042	0.7715	0.0278	14.0649	1.3840
runet	0.8469	0.0186	12.9054	0.9450	0.7895	0.0432	12.9054	1.3410
Aunet	0.8509	0.0164	14.8667	0.9109	0.7543	0.1045	14.8667	1.4419
daunet	0.8481	0.0155	14.6603	0.9105	0.7703	0.0311	14.6603	1.3604
d2unet	0.8447	0.0144	13.9448	0.9676	0.7790	0.0483	13.9448	1.4247
d2Aunet	0.8328	0.0456	14.5523	1.9611	0.7724	0.0351	14.5523	1.3411
r2unet	0.8390	0.0366	14.4749	1.0748	0.7788	0.0579	14.1360	1.3977
r2Aunet	0.8456	0.0152	13.1392	1.0938	0.7799	0.0365	13.1392	1.3406
vnet	0.8389	0.0172	12.4145	1.0663	0.7998	0.0282	12.4145	1.2589
segresnet	0.8234	0.0199	13.7308	1.2383	0.7729	0.0347	13.7308	1.3995
highresnet	0.8457	0.0186	15.0525	1.5373	0.8083	0.0212	15.0525	1.3386
nnunet	**0.8760**	0.0099	8.7049	1.2928	**0.8561**	0.0133	10.2390	1.1226
(**B**)								
	Tz				Cropped Tz			
	MDS	MHD (mm)		ASD (mm)	MDS	MHD (mm)		ASD (mm)
unet	**0.7700**	22.5822		2.4379	0.6424	16.8615		2.6838
unet++	0.7681	19.8427		2.3727	0.6536	16.9354		2.4165
runet	0.7671	19.9993		2.0027	0.6477	17.0520		2.5169
Aunet	0.7680	21.7833		2.4354	0.6577	16.0525		2.5444
daunet	0.7688	19.6406		2.4507	0.6471	17.4325		2.5915
d2unet	0.7627	21.2574		2.4447	0.6581	17.3590		2.7397
d2Aunet	0.7638	22.9369		2.3869	0.6581	16.7426		2.5895
r2unet	0.7631	19.8119		2.5560	0.6523	17.3374		2.8883
r2Aunet	0.7672	21.2282		2.1350	0.6553	14.3813		2.6186
vnet	0.7643	20.3670		2.8237	0.6603	18.4141		2.8833
segresnet	0.7381	19.6969		2.6089	0.6482	18.0439		2.7591
highresnet	0.7209	46.6032		6.4525	0.6381	22.3236		3.6603
nnunet	0.7300	16.9107		7.4144	**0.7540**	14.4592		2.1459

**Table 3:** (**A**) Results obtained on the external test set by the segmentation models trained on the peripheral zone segmentation task. The results presented are the mean scores of the 5-fold models over all the samples on the external test set. The best results (based on Table A3) for both the full and cropped volumes are presented in green. (**B**) Results obtained by the segmentation models on the peripheral zone segmentation task. The results presented are the mean scores from the validation data over the five folds. The best results for both the full and cropped volumes are presented in red.

(**A**)								
	Pz				Cropped Pz			
	MDS	CI	MHD (mm)	ASD (mm)	MDS	CI	MHD (mm)	ASD (mm)
unet	0.7545	0.0300	22.1193	1.5215	**0.7656**	0.0326	15.8711	1.1443
unet++	0.7557	0.0363	21.6545	1.4484	**0.7666**	0.0328	15.8122	1.1031
runet	0.7472	0.0296	23.0029	1.4938	**0.7627**	0.0243	15.8278	1.1482
aunet	0.7632	0.0280	19.6521	1.2725	**0.7611**	0.0360	15.0016	1.1684
daunet	0.7576	0.0384	20.1854	1.3109	**0.7655**	0.0349	15.1258	1.1131
d2unet	0.7597	0.0336	22.9687	1.4304	**0.7644**	0.0327	15.5292	1.0531
d2aunet	0.7614	0.0219	21.2204	1.5646	**0.7677**	0.0258	15.0999	1.1449
r2unet	0.7563	0.0280	24.6377	1.2007	**0.7700**	0.0307	15.4505	1.1609
r2aunet	0.7656	0.0294	19.6629	1.2778	**0.7689**	0.0343	14.8106	1.1195
vnet	0.7420	0.0315	22.0089	1.7818	**0.7573**	0.0297	15.9604	1.1659
segresnet	0.7329	0.0285	21.4516	1.4579	**0.7456**	0.0380	15.9265	1.2347
highresnet	0.7560	0.0301	35.7066	2.2781	**0.7719**	0.0203	18.4239	1.1279
nnunet	**0.8029**	0.0063	9.8693	1.0210	**0.7686**	0.0110	14.4054	1.1633
(**B**)								
	Pz				Cropped Pz			
	MDS		MHD (mm)	ASD (mm)	MDS		MHD (mm)	ASD (mm)
unet	0.6160		26.9297	2.7719	0.6217		21.3145	2.0339
unet++	0.6129		37.0787	3.5482	0.6064		19.8930	2.3919
runet	0.5828		19.5994	2.8543	0.6104		22.1387	2.4339
aunet	0.6224		20.8184	2.5188	0.6141		20.3642	2.5048
daunet	0.6282		23.0450	3.0015	0.6252		20.2531	2.0680
d2unet	0.6222		18.9975	2.5521	**0.6387**		16.3358	1.9674
d2aunet	0.6218		26.7974	2.6237	0.6372		17.8484	2.1356
r2unet	0.6273		27.9654	2.5934	0.6308		21.7236	2.2581
r2aunet	0.6228		21.5421	2.7400	0.6300		17.1794	2.0054
vnet	0.5848		40.8916	4.7720	0.5853		23.1373	2.5561
segresnet	0.6106		21.5214	2.5409	0.5339		41.5031	4.4359
highresnet	0.5669		60.2008	5.0504	0.6075		19.7673	2.8573
nnunet	**0.6835**		13.4527	1.8224	0.6038		17.6085	2.5589

## Data Availability

Data are available upon reasonable request.

## Appendix A

**Table A1:** *p*-values using Kruskal–Wallis for the dice scores obtained in the cross-validation of the gland segmentation task. On the top area we compare the performance of the different architectures on the full data, and on the bottom on the cropped data. On the diagonal we present the comparison of the same architecture on both data types. Significant values (p<0.05) are highlighted in green.

Gland	unet	unet++	d2unet	d2aunet	runet	aunet	daunet	r2unet	r2aunet	segresnet	highresnet	vnet	nnunet	
unet	0.6015	0.3472	0.9168	0.7540	0.9168	0.7540	0.6015	0.7540	0.9168	0.1745	0.1745	0.2506	**0.009**	
unet++	0.4647	0.9168	0.4647	0.3472	0.9168	0.7540	0.7540	0.9168	0.9168	0.0758	0.1172	0.1172	**0.009**	
d2unet	0.1172	**0.0283**	0.3472	0.7540	0.9168	0.7540	0.4647	0.9168	0.6015	0.0758	0.2506	0.1172	**0.009**	
d2aunet	0.1172	**0.0283**	0.9168	0.6015	0.6015	0.2506	0.4647	0.4647	0.6015	**0.0472**	0.0758	**0.0472**	**0.009**	
runet	0.7540	0.6015	0.0758	0.0758	0.9168	0.7540	0.7540	0.7540	0.7540	**0.0472**	0.1172	**0.0472**	**0.009**	
aunet	0.6015	0.6015	0.6015	0.2506	0.6015	0.3472	0.9168	0.7540	0.7540	0.3472	0.3472	0.3472	**0.009**	
daunet	0.6015	0.2506	0.6015	0.4647	0.4647	0.6015	0.2506	0.7540	0.6015	0.3472	0.4647	0.4647	**0.009**	
r2unet	0.4647	0.6015	0.7540	0.3472	0.4647	0.7540	0.7540	0.2506	0.9168	**0.0472**	0.1745	0.0758	**0.009**	Full
r2aunet	0.2506	**0.0283**	0.6015	0.4647	0.1172	0.6015	0.7540	0.9168	0.4647	0.1745	0.2506	0.1745	**0.009**	
segresnet	0.2506	0.1172	**0.0163**	**0.0163**	0.2506	0.1172	**0.0283**	**0.0283**	**0.0163**	0.1172	0.9168	0.7540	**0.009**	
highresnet	0.1172	0.0758	**0.0163**	**0.0163**	0.1745	0.1172	**0.0283**	0.1172	**0.0163**	0.9168	0.4647	0.7540	**0.009**	
vnet	0.3472	0.3472	**0.0472**	**0.0472**	0.4647	0.4647	0.1172	0.2506	0.0758	0.3472	0.4647	**0.0472**	**0.009**	
nnunet	0.1745	**0.0283**	0.6015	0.6015	0.0758	0.4647	0.6015	0.9168	0.7540	**0.0163**	**0.0163**	**0.0472**	**0.009**	
	Cropped													full vs. cropped

**Table A2:** *p*-values using Kruskal–Wallis for the dice scores obtained in the cross-validation of the transition segmentation task. On the top area we compare the performance of the different architectures on the full data, and on the bottom on the cropped data. On the diagonal we present the comparison of the same architecture on both data types. Significant values (p<0.05) are highlighted in green.

TZ	unet	unet++	d2unet	d2aunet	runet	aunet	daunet	r2unet	r2aunet	segresnet	highresnet	vnet	nnunet	
unet	**0.009**	0.7540	0.7540	0.4647	0.4647	0.2506	0.7540	0.7540	0.7540	0.1172	0.9168	0.7540	**0.0163**	
unet++	0.7540	**0.009**	0.6015	0.6015	0.9168	0.7540	0.9168	0.4647	0.9168	0.1172	0.9168	0.4647	**0.009**	
d2unet	0.7540	0.6015	**0.009**	0.3472	0.9168	0.6015	0.7540	0.6015	0.7540	**0.0472**	0.9168	0.3472	**0.009**	
d2aunet	0.4647	0.6015	0.3472	**0.0163**	0.2506	0.2506	0.6015	0.9168	0.6015	0.4647	0.6015	0.7540	**0.0163**	
runet	0.4647	0.9168	0.9168	0.2506	**0.0472**	0.3472	0.7540	0.6015	0.7540	**0.0472**	0.7540	0.1745	**0.0163**	
aunet	0.2506	0.7540	0.6015	0.2506	0.3472	**0.0163**	0.7540	0.3472	0.6015	**0.0472**	0.7540	0.1745	**0.0163**	
daunet	0.7540	0.9168	0.7540	0.6015	0.7540	0.7540	**0.009**	0.3472	0.4647	0.1172	0.7540	0.4647	**0.009**	
r2unet	0.7540	0.4647	0.6015	0.9168	0.6015	0.3472	0.3472	**0.0283**	0.6015	0.4647	0.3472	0.9168	**0.009**	Full
r2aunet	0.7540	0.9168	0.7540	0.6015	0.7540	0.6015	0.4647	0.6015	**0.009**	0.0758	0.7540	0.3472	**0.009**	
segresnet	0.1172	0.1172	**0.0472**	0.4647	**0.0472**	**0.0472**	0.1172	0.4647	0.0758	**0.0163**	0.1172	0.1745	**0.009**	
highresnet	0.9168	0.9168	0.9168	0.6015	0.7540	0.7540	0.7540	0.3472	0.7540	0.1172	**0.0283**	0.7540	**0.009**	
vnet	0.7540	0.4647	0.3472	0.7540	0.1745	0.1745	0.4647	0.9168	0.3472	0.1745	0.7540	**0.0472**	**0.009**	
nnunet	**0.0163**	**0.009**	**0.009**	**0.0163**	**0.0163**	**0.0163**	**0.009**	**0.009**	**0.009**	**0.009**	**0.009**	**0.009**	**0.0283**	
	Cropped													full vs. cropped

**Table A3:** *p*-values using Kruskal–Wallis for the dice scores obtained in the cross-validation of the peripheral segmentation task. On the top area we compare the performance of the different architectures on the full data, and on the bottom on the cropped data. On the diagonal we present the comparison of the same architecture on both data types. Significant values (p<0.05) are highlighted in green.

PZ	unet	unet++	d2unet	d2aunet	runet	aunet	daunet	r2unet	r2aunet	segresnet	highresnet	vnet	nnunet	
unet	0.4647	0.6015	0.9168	0.6015	0.3472	0.4647	0.9168	0.9168	0.3472	0.1745	0.6015	0.1745	**0.0163**	
unet++	0.9168	0.4647	0.9168	0.1745	0.4647	0.3472	0.7540	0.6015	0.1745	0.1172	0.3472	0.2506	**0.0283**	
d2unet	0.9168	0.9168	0.9168	0.6015	0.3472	0.6015	0.9168	0.7540	0.2506	0.0758	0.7540	0.2506	**0.0163**	
d2aunet	0.9168	0.9168	0.9168	0.4647	0.0758	0.7540	0.4647	0.2506	0.4647	0.0758	0.7540	0.0758	**0.0163**	
runet	0.7540	0.6015	0.9168	0.6015	0.2506	0.1745	0.4647	0.3472	0.1172	0.2506	0.3472	0.4647	**0.009**	
aunet	0.7540	0.6015	0.9168	0.6015	0.9168	0.9168	0.4647	0.3472	0.7540	0.0758	0.7540	0.1745	**0.0163**	
daunet	0.9168	0.7540	0.9168	0.7540	0.6015	0.6015	0.6015	0.9168	0.3472	0.1172	0.4647	0.3472	**0.0283**	
r2unet	0.7540	0.9168	0.7540	0.9168	0.9168	0.6015	0.7540	0.3472	0.2506	0.0758	0.3472	0.1745	**0.0163**	Full
r2aunet	0.9168	0.9168	0.6015	0.9168	0.7540	0.7540	0.9168	0.7540	0.7540	0.0758	0.6015	0.0758	**0.0163**	
segresnet	0.2506	0.1745	0.2506	0.1745	0.3472	0.4647	0.3472	0.1745	0.2506	0.3472	0.3472	0.3472	**0.009**	
highresnet	0.9168	0.9168	0.7540	0.6015	0.3472	0.3472	0.4647	0.9168	0.7540	0.1172	0.3472	0.3472	**0.0283**	
vnet	0.4647	0.4647	0.6015	0.4647	0.6015	0.7540	0.4647	0.4647	0.4647	0.6015	0.2506	0.1745	**0.009**	
nnunet	0.9168	0.7540	0.7540	0.7540	0.4647	0.6015	0.9168	0.9168	0.7540	0.1745	0.6015	0.2506	**0.0163**	
	Cropped													full vs. cropped
